# Research on the Adhesion Properties of Fast-Melting SBS-Modified Asphalt–Aggregate Based on Surface Free Energy Theory

**DOI:** 10.3390/ma16247601

**Published:** 2023-12-11

**Authors:** Bo Men, Fei Guo, Xiaolong Kang, Jinchao Yue

**Affiliations:** 1 Henan Xuxin Expressway Co., Ltd., Zhumadian 463000, China; menbo111@163.com; 2 School of Water Conservancy and Transportation, Zhengzhou University, Zhengzhou 450001, China; 17513361957@163.com (F.G.); zzukangxiaolong@163.com (X.K.)

**Keywords:** fast-melting SBS modifier, surface free energy, cohesion work, adhesion work, stripping work, energy ratio

## Abstract

In order to study the adhesion properties of fast-melting SBS-modified asphalt (SBS-T) at the interface with aggregates, the contact angles of three dosages of SBS-T asphalt with three liquids (distilled water, glycerol, and formamide) were determined by the sessile drop method based on surface free energy theory. The evaluation indexes such as cohesion, asphalt–aggregate adhesion, stripping work and energy ratio of the asphalt were analyzed and the adhesion properties of the asphalt–aggregate system were investigated with the help of micromechanical methods. The results indicate that SBS-T can improve the adhesion properties of the asphalt. Furthermore, as the dosage of the modifier increases, the cohesion work, adhesion work, and energy ratio of the SBS-T asphalt exhibit a similar rise. As the spalling work reduces and the adhesion between asphalt and aggregate improves, it is noteworthy that the SBS-T asphalt–aggregate system exhibits superior adhesion performance compared to the SBS-modified asphalt–aggregate system, despite the same dosage.

## 1. Introduction

With the development of highway construction, vehicle overloading and canalized traffic are becoming increasingly severe. Under vehicle load, water enters the asphalt pavement, which will cause damage to the asphalt surface layer, such as pitting, loosening, raveling, kipping, network cracks, and potholes. These defects significantly impact driving comfort and traffic safety. Therefore, it is critical to employ high-performance modified asphalt components to increase asphalt pavement’s resistance to water damage [1,2].

High-viscosity and high-elasticity asphalt can enhance the adhesion to the aggregate. Therefore, this type of asphalt pavement has good resistance to water damage. It has many applications in the drainage of pavements [3]. Currently, high-viscosity and high-elasticity modified asphalt prepared by traditional processes has the problem of unstable thermodynamic properties. The root of the problem is the traditional preparation process. The conventional process involves using high-speed shears to break down SBS and mix it with asphalt as a solution. However, this process has problems such as low efficiency and high energy consumption. Moreover, SBS will precipitate after being incorporated into asphalt, and this precipitation will be more serious with the longer storage time. High temperatures during transportation can lead to deterioration of the modified bitumen and structural damage to the SBS. The preparation of modified asphalt using conventional modification processes requires a continuous high temperature shear process [4]. Maintaining high temperatures requires a lot of energy and produces a lot of greenhouse gases, which puts a lot of pressure on ecosystems [5,6,7]. In order to avoid these problems at the source, we can use the fast melting process for modification. The key to the fast-melting modification process is to shorten the melting time of the modifier and to speed up the dissolution of the modifier in the asphalt [8]. Based on this, Guolu Hi-Tech optimized the structure of the SBS modifier, added components to enhance the quick-melting ability to achieve a balance between the melt index and modification effect, and prepared a quick-melting SBS modifier (SBS-T); the melt index is 100 times higher than that of ordinary SBS, and it can be used directly after being added to the mixture, realizing 1 min rapid melting [9]. Due to the problems of performance degradation in traditional modified asphalt, the addition of the SBS-T modifier can improve this phenomenon. The SBS-T modifier is fed into the mixing plant, where it can be mixed directly with the aggregate to produce a modified asphalt mixture. It can avoid short-term aging at high temperatures. Thus, it can overcome the problems of traditional wet SBS-modified asphalt materials in the construction process.

The application of a fast-melting SBS modifier as a new type of modifier needs to be further verified. Only a limited number of current studies analyze adhesion properties between SBS-T asphalt and aggregates. The most widely used technique to evaluate the adhesion quality between asphalt and aggregate is the water boiling method. However, the influence of human factors is too significant to analyze the adhesion of asphalt–aggregate systems quantitatively. In recent years, surface free energy theory is commonly used as an evaluation of the adhesion properties between asphalt and aggregate. In the field of road engineering, surface free energy refers to the energy required when the asphalt–aggregate system increases the unit surface area under the condition of constant temperature and pressure. Surface energy theory reveals the asphalt–aggregate adhesion and spalling phenomena through the perspective of energy change. The adhesion of asphalt–aggregate is modeled on a microscopic scale. It can not only reveal the damage mode of asphalt–aggregate and the water damage mechanism, but also evaluate the water stability of asphalt mixtures. It is of great significance to analyze the influencing factors of asphalt–aggregate adhesion performance and improve the water damage resistance of asphalt pavement. With the development of thermodynamic surface energy theory, many scholars have used this theory to evaluate the adhesion properties of asphalt–aggregate systems. Han et al. [10] constructed an asphalt–aggregate adhesion damage model on the basis of surface free energy theory. The results showed that changes in the adhesion properties of asphalt–aggregate systems can be reflected by adhesion work. Howson et al. [11,12] evaluated an asphalt–aggregate system’s adhesion properties in both the dried state and with the presence of water. Bhasin et al. [13] introduced two energy ratio (ER) parameters as evaluation indexes based on the work of adhesion and work of exfoliation in order to evaluate surface energy adhesion properties. ER_1_ considers the work of adhesion in the presence and absence of water. ER_2_ considers the work performed by the wettability of the bitumen and the cohesion of the aggregate. Li et al. [14] compared the thermodynamic parameters with freeze–thaw cracking test data. The results show that the water damage resistance of asphalt mixtures can be reflected visually by the spalling work index. In this paper, the adhesion properties between SBS-T-modified asphalt and aggregates (limestone, granite) were investigated based on surface free energy theory. The sessile drop method testing technique and surface energy theory were used to calculate thermodynamic parameters such as cohesion work, adhesion work, exfoliation work, and energy ratio, which provide the theoretical basis for applying SBS-T asphalt in the drainage of pavements.

## 2. Theoretical Background

The energy required for materials to separate or form new interfaces is known as surface free energy. From the viewpoint of the change in interfacial energy, it primarily explains the phenomenon of adhesion and the spalling of asphalt and aggregate [15]. The GvOC model is the most commonly used surface energy model of road asphalt materials. According to the theory, polar and nonpolar components can be distinguished in a solid material’s surface free energy. Polar components include two types: Lewis acidic and Lewis alkaline, as given in Equation (1).

(1) $$\gamma^{total}=\gamma^{LW}+\gamma^{AB}=\gamma^{LW}+2\sqrt{\gamma^{+}\gamma^{-}}$$

where $\gamma^{total}$ is the total surface energy of the material. $\gamma^{LW}$ is the nonpolar dispersive component of the surface energy. $\gamma^{AB}$ is the polar component (acid-base component). $\gamma^{+}\;$ is the Lewis acidic component. $\gamma^{-}$ is the Lewis basic component.

There is a link between the contact angle of liquid and the surface energy of solid asphalt, as can be expressed by Equation (2).

(2) $$\gamma_{s}=\gamma_{sl}+\gamma_{l}\cos\theta$$

where $\gamma_{s}$ is the solid surface energy. $\gamma_{l}$ is the surface energy of the liquid. $\gamma_{sl}$ is the interfacial free energy between solid and liquid.

The energy required to separate the asphalt–aggregate interface in the waterless state is called the asphalt–aggregate adhesion work. The value of the work of adhesion can be calculated according to Equation (3).

(3) $$W_{AB}=2\sqrt{\gamma_{A}^{LW}\gamma_{B}^{LW}}+2\sqrt{\gamma_{A}^{+}\gamma_{B}^{-}}+2\sqrt{\gamma_{A}^{-}\gamma_{B}^{+}}$$

where *A* is asphalt and *B* is aggregate.

Based on thermodynamics, the energy required to separate the uniform asphalt material into two new interfaces is defined as the cohesion work of the asphalt. Its calculation formula is shown in Equation (4).

(4) $$W_{BB}=2\gamma_{B}$$

The energy released when asphalt is replaced by water at the asphalt–aggregate interface results in the spalling work. Its magnitude can be expressed by Equation (5).

(5) $$\begin{array}{c} W_{aBWA}^{wet}=2\gamma_{W}^{LW}+2\sqrt{\gamma_{B}^{LW}\gamma_{W}^{LW}}-2\sqrt{\gamma_{A}^{LW}\gamma_{W}^{LW}}\\ +4\sqrt{\gamma_{W}^{+}\gamma_{W}^{-}}-2\sqrt{\gamma_{W}^{+}}\left(\sqrt{\gamma_{B}^{-}}+\sqrt{\gamma_{A}^{-}}\right)\\ -2\sqrt{\gamma_{W}^{-}}\left(\sqrt{\gamma_{B}^{+}}+\sqrt{\gamma_{A}^{-}}\right)+2\sqrt{\gamma_{B}^{+}\gamma_{A}^{-}}\\ +2\sqrt{\gamma_{B}^{-}\gamma_{A}^{+}} \end{array}$$

where *w* is water.

Bhasin et al. [16] suggested using the energy ratio parameter, which is determined by the ratio of the work of adhesion in the dry state to the work of spalling in the presence of water. This approach has been based on numerous experimental studies and is considered a reliable way to evaluate the water stability of asphalt mixtures. Larger energy ratio values correspond to increased resistance to water damage.

(6) $$ER=\frac{W_{AB}}{W_{aBAW}^{wet}}$$

## 3. Materials and Methodologies

### 3.1. Preparation of Quick-Melting SBS-Modified Asphalt

#### 3.1.1. Matrix Asphalt

In this paper, 70# asphalt, commonly used in road construction, is adopted as the base asphalt to prepare SBS- and SBS-T-modified asphalts. According to the test method in JTG E20-2011 “Technical Specification for Highway Asphalt Pavement Construction” [17], the relevant test of asphalt technical indicators was carried out. Its basic technical properties are listed in Table 1.

#### 3.1.2. Asphalt Modifier

**Figure 1:**
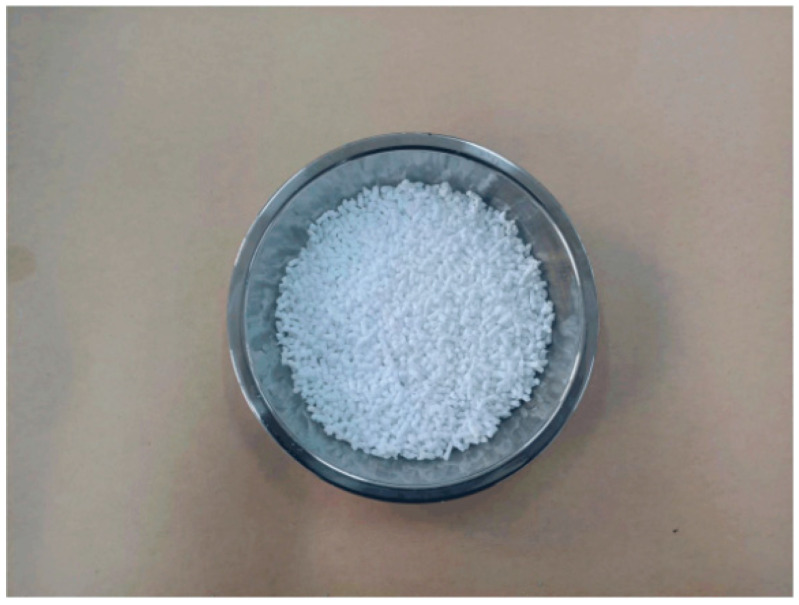
SBS modifier.

**Figure 2:**
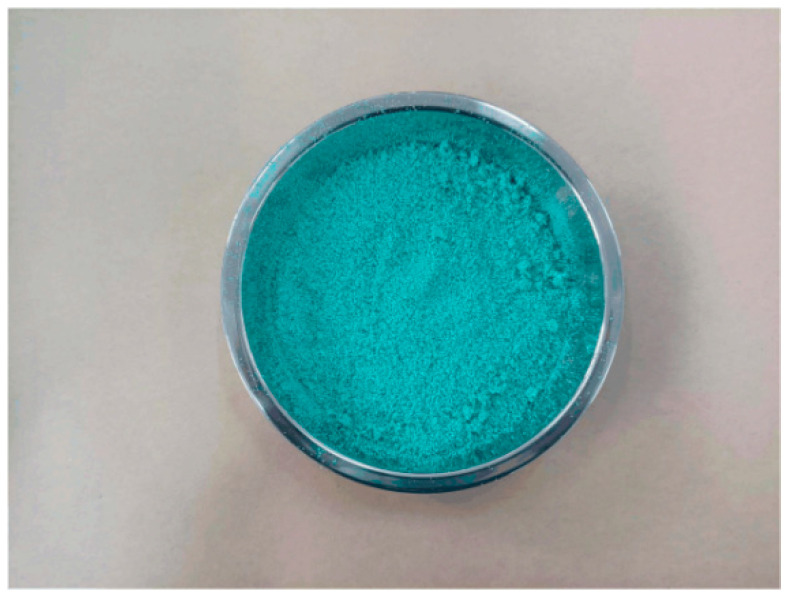
SBS-T modifier.

In this study, Guolu Gaoke Engineering Technology Institute Co., Ltd. (No. 30 College Rd., Haidian District, Beijing, China) offered the SBS and SBS-T modifiers. The pictures are shown in Figure 1 and Figure 2. Their fundamental technical indicators are shown in Table 2.

#### 3.1.3. Modified Asphalt Preparation

The asphalt was first heated in the oven at 150 °C to a molten state. Subsequently, a certain mass of the SBS-T modifier was used in the base asphalt. At the same time, a glass rod was used to mix the asphalt and SBS-T. The shearing process was carried out using an FLUKO FM300 high-speed shear machine. The manufacturer of the machine is FLUKO Shanghai Equipment Co., Ltd. (Shanghai, China). The machine was used to shear the asphalt at a speed of 5000 r/min for 30 min. The temperature of the shearing process was 180 °C. In this way, SBS-T-modified asphalt with mass fractions of 5%, 6.5%, and 8% can be prepared.

This study describes the testing of three different dosages of high-viscosity SBS-T-modified asphalt for the assessment of penetration, softening points, ductility, dynamic viscosity at 60 °C, and elasticity recovery. Table 3 displays the obtained results, showing that different dosages have fulfilled the technical criteria for high-viscosity and high-elasticity modified asphalt.

### 3.2. Contact Angle Measurement Methods

#### 3.2.1. Test Method

The size of the contact angle is related to the wettability of the solvent on asphalt. When the contact angle is less than 90°, it indicates that the solvent can wet the asphalt surface. When the contact angle is greater than 90°, it indicates that the solvent and asphalt have poor wettability. When the contact angle is equal to 0° or 180°, it indicates that the test liquid completely wets or does not wet the asphalt surface.

**Figure 3:**
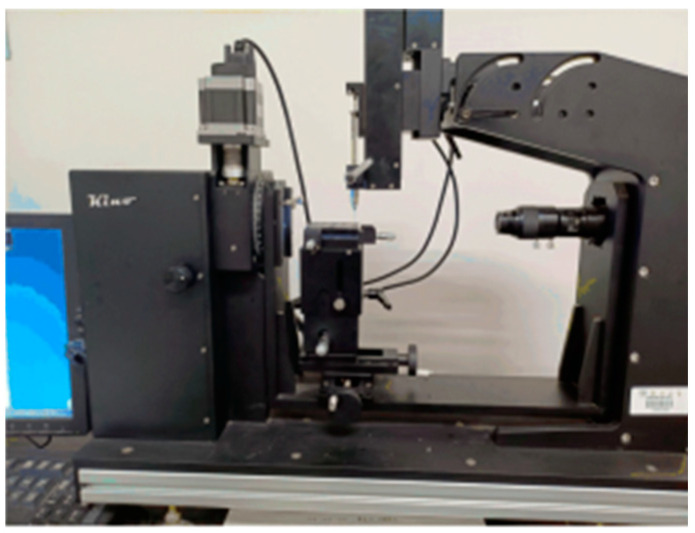
Optical contact angle measuring instrument.

This experiment used the SL200KS optical contact angle and interface tension measuring instrument to measure the contact angle of the sample, which includes a light source system, a video acquisition system, and an analysis system. The manufacturer of the optical contact Angle measuring instrument is SOLON TECH (Shanghai, China), as shown in Figure 3. The main principle is to capture the contact process between the test liquid and the sample using a high-resolution camera before then locking the screen into a static image using contact angle analysis software CAST 2.0. The static contact angle is obtained using a circle fitting method.

When using the sessile drop method to determine the contact angle of a sample, the basic assumptions are as follows: the droplets are vertically symmetrical and the shape of a droplet is only related to its own interfacial tension and gravity. When selecting testing liquids, the following principles should be met: (1) the surface energy of the liquid should be greater than the surface energy of the solid to be tested; (2) there should be no chemical reaction between the liquid and the solid to be tested; (3) liquids should be non-toxic.

In this investigation, contact angle measurements were based on the sessile drop method. The liquids used for the test included distilled water, glycerol, and formamide. To prevent interference between liquids, three samples of each asphalt were prepared, and one sample was used for each liquid. In order to check the accuracy of the results, five measurements were made on each specimen. The average of the five measurements was taken. The study was carried out using limestone and granite. The contact angles between the aggregates and three different liquids (distilled water, glycerol, and formamide) were determined using the sessile drop method. Subsequently, the surface energy of the aggregate and its components was calculated based on Equation (2), as shown in Table 4. Since the sessile droplet method is carried out in a normal environment, the aggregate surface adsorbs moisture from the air and impurity molecules, causing the measured surface energy of the aggregate to be too small. However, the results of the measurements were relatively close to the data of previous studies [18,19], proving the feasibility of test results. The surface energy of asphalt materials and their fractions are significantly lower than those of aggregates. This is because aggregates belong to objects with high surface energy, and their surfaces will adsorb with asphalt by reducing their surface energy [20].

#### 3.2.2. Asphalt Sample Preparation

When measuring the contact angle, three solutions of distilled water, glycerol, and methylamine should be used to titrate on a smooth and pollution-free asphalt surface, and the contact angle of the test solution on the asphalt surface should then be measured using a contact angle meter. Therefore, the asphalt contact angle sample should ensure that the asphalt surface is smooth and flat.

**Figure 4:**
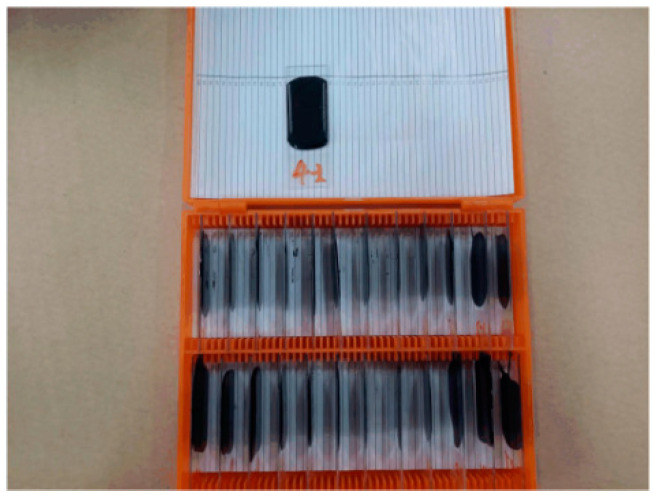
Contact angle samples of asphalt binders.

The preparation method for asphalt samples used in this article is as follows: Firstly, the prepared asphalt samples were heated to flow conditions. Then, a little bit of asphalt was applied to the slide with a glass rod. The asphalt was heated at 150 °C for 20 min. At the same time, the slides were treated in the same way. The asphalt became free-flowing and formed a smooth surface. The asphalt samples were then cooled by placing them in a 25 °C environment (room temperature). Finally, the cooled samples were put in a desiccator to wait for testing. The samples are indicated in Figure 4.

#### 3.2.3. Aggregate Sample Preparation

Aggregate plays an important role in evaluating the adhesion properties between asphalt and aggregate [21]. The current methods for testing the surface energy of aggregates mainly include the contact angle method, the heat method, and the adsorption method. 

**Figure 5:**
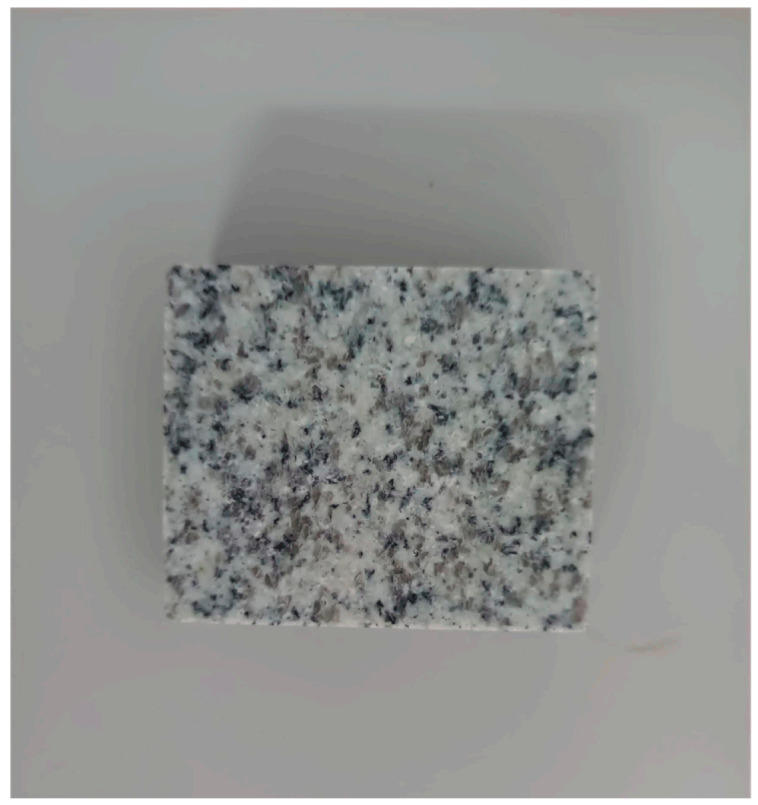
Aggregate contact angle sample.

To prepare the aggregate samples, the stone was first cut into rectangular squares of 60 mm × 40 mm × 15 mm using a cutting machine. It was then polished with an angle grinder and sanded to a smooth surface with 1200 grit and 600 grit sandpaper. Finally, the aggregate surface was cleaned with alcohol and distilled water to prevent the rough surface and dust from affecting the contact angle measurement results of the aggregate sample. An aggregate contact angle sample is shown in Figure 5.

## 4. Results and Analysis

### 4.1. Cohesive Work

**Figure 6:**
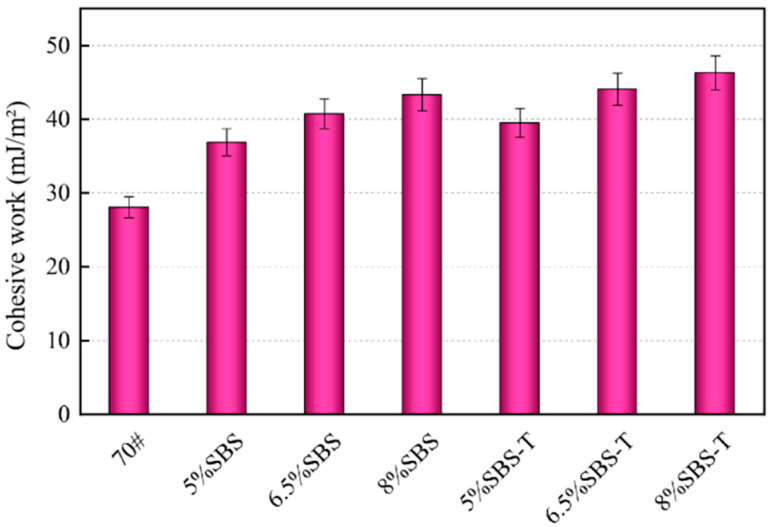
Cohesive work of SBS and SBS-T asphalt binders.

Higher values of cohesiveness correspond to higher resistance to cracking of the asphalt, making it harder for water to enter the asphalt–aggregate surface through the cracks [22]. The cohesive work of three dosages of SBS and SBS-T asphalt is shown in Figure 6.

Figure 4 indicates that the cohesive energy of SBS-modified asphalt ranges from 36.91 to 42.83 mJ/m^2^, while for SBS-T asphalt, it ranges from 38.95 to 45.20 mJ/m^2^. The cohesive properties of both types of modified asphalt increase as the amount of modifier is increased. When adding the same amount of doping, the cohesive work of SBS-T asphalt is higher. This suggests that SBS-T asphalt exhibits superior resistance to cracking. This may be because the SBS-T modifier can achieve micron-level dispersion. Its physical structure is only 1/100th of that of the SBS modifier, while its melt index is more than 100 times higher than that of the SBS modifier. Consequently, it can melt quickly and completely with the matrix asphalt. There is no compatibility problem after melting SBS-T and matrix asphalt to form more physical crosslinks and entanglement. This improves the embedding force between the SBS-T modifier and the asphalt. This, in turn, increases bitumen adhesion by creating a more stable three-dimensional network structure.

### 4.2. Adhesion Work

**Figure 7:**
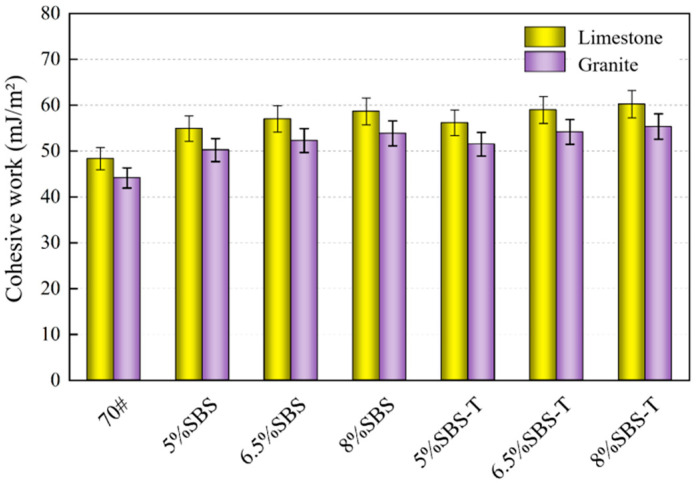
Adhesion work of SBS and SBS-T asphalt binders to aggregates.

Higher adhesion indicates a more stable asphalt–aggregate system. Its value is linked to the surface energy of both components, as well as the interface energy of the asphalt–aggregate. The adhesion work can be calculated using Equation (3), with the resulting calculations depicted in Figure 7.

In Figure 7, in the system consisting of asphalt and limestone, the adhesion work of SBS-modified asphalt was improved by 13.6%, 17.9%, and 21.3% for dosages of 5%, 6.5%, and 8%, respectively. The same dosage of SBS-T-modified asphalt increased the adhesion works by 16.2%, 22%, and 24.5%, respectively. SBS-T-modified asphalt exhibits superior adhesion performance to SBS-modified asphalt with the same aggregate. The increased viscosity of SBS-T-modified asphalt may be due to the adsorption of light components, such as dissolved, saturated, and aromatic components, by the SBS-T modifier. Existing studies indicate that there is a correlation between asphalt viscosity and the adhesion between asphalt and aggregate. Consequently, by using high-viscosity SBS-T-modified asphalt and an aggregate, a more stable system can be formed. However, it is worth noting that when the SBS-T modifier dosage reaches 6.5%, the improvement in adhesion work is insignificant. This may be due to an excessive amount of SBS-T modifier. Excess modifier will lead to a large amount of light oil being absorbed by the modified asphalt, including components that result in reductions in adhesion to the aggregate.

Based on research conducted by Grenfell et al. [23], the bond between modified asphalt and limestone is stronger compared to that with granite due to the surface energy of the aggregate. Since limestone has a higher surface energy, the adhesion to asphalt is stronger, a conclusion that is consistent with that reached in this study. Limestone aggregate is alkaline with high calcite content, and granite is acidic with high silica content. The asphalt is weakly acidic, and the bonding between limestone and asphalt is mainly realized by chemisorption. Physical adsorption is responsible for the bonding between granite and asphalt. Therefore, the system of limestone and asphalt has better adhesion properties and is more stable [24]. In addition, highly acidic asphalt and highly alkaline aggregates correspond to the asphalt mixtures with high water damage resistance.

### 4.3. Stripping Work

**Figure 8:**
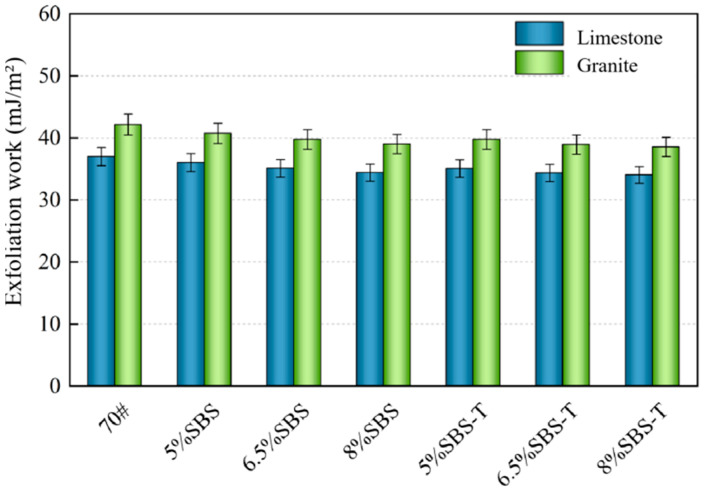
Stripping work of SBS and SBS-T asphalt binders to aggregates.

Moisture is the root cause of adhesion failure in asphalt–aggregate systems, which occurs when moisture enters the interior of an asphalt mixture through cracks or vehicle loading. As the aggregate binds more readily to the polarized water, this causes the asphalt to spall off the top of the aggregate. The energy released to the outside world by this process is known as the work of spalling. The spalling works of different modified asphalt and two aggregates (limestone and granite) are shown in Figure 8.

Figure 8 shows that the addition of two modifiers resulted in varying degrees of reduction in stripping work. When using limestone aggregate, SBS-modified asphalt had a stripping work range of 34.40–36.02 mJ/m^2^, while the stripping work of SBS-T asphalt ranged from 34.03 to 35.04 mJ/m^2^. As for the granite aggregate, the stripping work was 39.74–38.53 mJ/m^2^ for SBS-T asphalt and 40.72–39.00 mJ/m^2^ for SBS-modified asphalt. This suggests that SBS and SBS-T can reduce the effect of water on asphalt and favor asphalt adhesion properties. The SBS-modified asphalt is weaker than SBS-T-modified asphalt in terms of resisting water damage. The spalling work of the same aggregate with different modified asphalt can be summarized as follows: SBS-T-modified asphalt < SBS-modified asphalt. For SBS- and SBS-T-modified asphalt, the order of exfoliation work with different aggregates is as follows: limestone < granite. Granite is an acidic aggregate, and the surface contains polar groups with hydrophilic properties, which more easily combine with water molecules and cause asphalt spalling. In addition, the granite surface is relatively smooth, and the area covered by asphalt is much smaller than that of alkaline stone. Therefore, it is easily affected by water.

### 4.4. Energy Ratio

**Figure 9:**
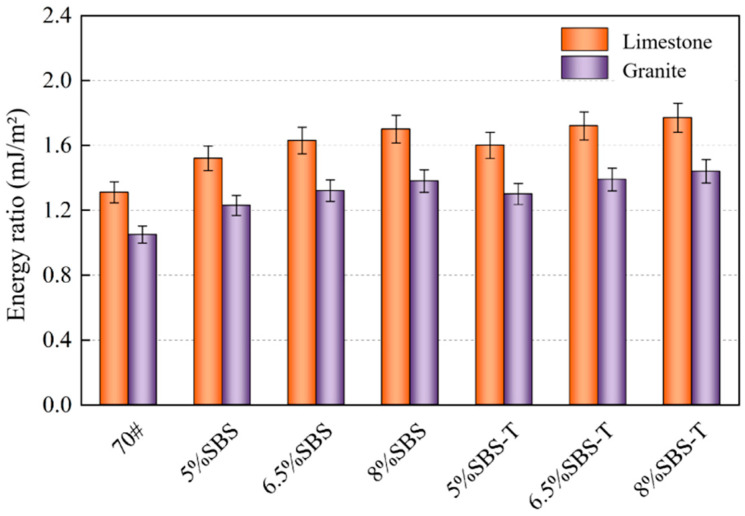
Energy ratio of SBS and SBS-T asphalt binders to aggregates.

According to Equation (6), the ratio of the work of adhesion of asphalt in the absence of water and the work of spalling between asphalt and aggregate in the presence of water is defined as an energy ratio. The energy ratio of asphalt to aggregate is indicated in Figure 9.

Figure 9 suggests that, in the system consisting of asphalt and limestone compared to the base asphalt, the addition of 5%, 6.5%, and 8% of SBS and SBS-T modifiers to the asphalt limestone aggregate system increased the energy coefficient of SBS-T-modified asphalt by 22.4%, 31.1%, and 35.1% and the energy ratio of SBS-modified asphalt by 16.4%, 24%, and 30%, respectively. In the system consisting of asphalt and granite, the energy ratios of SBS-modified asphalt ranged from 1.23 to 1.38, while for SBS-T asphalt, it ranged from 1.30 to 1.44. Based on the preceding analysis, SBS-T asphalt exhibits greater adhesion work than SBS-modified asphalt, regardless of whether limestone or granite is used. Furthermore, the absolute value of stripping work is smaller for SBS-T than for SBS-modified asphalt. Therefore, SBS-T asphalt has a higher energy ratio than SBS-modified asphalt, and higher SBS-T content corresponds to a higher energy ratio. This indicates that SBS-T asphalt has better adhesion to the aggregate, with 8% SBS-T asphalt having the most substantial resistance to water damage.

## 5. Conclusions

This study is based on the micro mechanical method and takes high-viscosity SBS-T-modified asphalt prepared with a new type of instant modifier as the research object. The adhesion properties between high-viscosity SBS-T-modified asphalt and limestone and granite were investigated. In addition, in order to measure and analyze the contact angle between asphalt and aggregate and calculate the surface energy of the aggregate and its components, this paper is based on experiments conducted using the sessile drop method. Surface free energy theory allows for the thermodynamic parameters of asphalt and aggregates to be calculated, and the key findings are as follows:

1. The cohesive energy of SBS-modified asphalt ranges from 36.91 to 42.83 mJ/m^2^, while for SBS-T asphalt, it ranges from 38.95 to 45.20 mJ/m^2^. This indicates that the addition of SBS-T had a significant positive impact on the thermomechanical properties of asphalt. As modifier dosage was increased, a more stable network structure was formed within the asphalt by the modifier. The cohesion work of asphalt also increased, leading to enhanced internal resistance against the cracking of asphalt material.
2. For the limestone aggregate, the stripping work of SBS-modified asphalt ranges from 34.40 to 36.02 mJ/m^2^, and that of SBS-T-modified asphalt ranges from 34.03 to 35.04 mJ/m^2^. The stripping work of SBS-T-modified asphalt ranges from 39.74 to 38.53 mJ/m^2^, and that of SBS-modified asphalt ranges from 40.72 to 39.00 mJ/m^2^. As the dosage of SBS-T modifier increases, the adhesion work and energy ratio of the asphalt–aggregate system increases and the spalling work reduces. The best adhesion of SBS-T-modified asphalt and aggregate is achieved when the dosage reaches 8%, which corresponds to the strongest resistance to water damage within the asphalt and aggregate system.
3. By analyzing the adhesion work, stripping work, and energy ratio of different asphalt–aggregate systems, the adhesion properties of different types of asphalt to aggregates can be ranked in the following order: SBS-T-modified bitumen > SBS-modified bitumen > matrix asphalt. The adhesion properties of the same bitumen to different aggregates can be ranked in the following order: limestone > granite. Among several combinations, the system consisting of SBS-T-modified asphalt and limestone has the best water resistance.
4. In this study, the contact angle of the samples was measured using the lay drop method based on the micromechanical scale. The thermodynamic parameters of asphalt and aggregate were calculated using surface free energy theory. A method of evaluating the adhesion properties between new modified asphalt and aggregate at the microscopic scale was thus obtained.

## Figures and Tables

**Table 1:** Basic technical specifications for 70# asphalt.

Technical Indicators	Unit	Measured Value	Test Methods ^a^
Penetration (25 °C)	0.1 mm	66.9	T0604-2011
Ductility (15 °C, 5 cm/min)	cm	>100	T0605-2011
Softening point	°C	53.2	T0606-2011
Brinell rotational viscosity (135 °C)	Pa·s	0.61	T0625-2011

^a^ The test methods are in accordance with JTG E20-2011.

**Table 2:** Technical specifications of modifiers.

Modifier	Technical Indicator	Unit	Measured Value
SBS	Structure type	-	Linearity
	Block ratio S/B	-	40/60
	Fracture elongation	%	≥700
	Melt flow rate	g/10 min	0.1–5.0
SBS-T	Appearance	-	Granular, uniform
	SBS content	%	≥50
	Ash content	%	≤5.0
	Melt index	g/10 min	≥2.0
	Dry mix dispersibility	-	No particle residue

**Table 3:** Technical specifications of SBS-T-modified asphalt binders.

Technical Indicator	Unit	5% SBS-T	6.5% SBS-T	8% SBS-T
Penetration (25 °C)	0.1 mm	61.8	58.6	56.3
Softening point	°C	88.7	93.2	95.1
Ductility (15 °C, 5 cm/min)	cm	42.5	47.9	50.4
Dynamic viscosity at 60 °C	Pa·s	27,096	68,252	134,527
Elastic recovery	%	96.7	99.1	99.5

**Table 4:** Surface free energy of aggregates and the components.

Aggregate Type	Unit	$\gamma_{l}$	$\gamma_{l}^{LW}$	$\gamma_{l}^{AB}$	$\gamma_{l}^{+}$	$\gamma_{l}^{-}$
limestone	mJ/m^2^	42.17	32.72	9.45	1.55	14.39
granite	mJ/m^2^	35.51	26.96	8.55	1.67	10.92

## Data Availability

Data are contained within the article.
